# Machine Learning Approaches to Predict Risks of Diabetic Complications and Poor Glycemic Control in Nonadherent Type 2 Diabetes

**DOI:** 10.3389/fphar.2021.665951

**Published:** 2021-06-22

**Authors:** Yuting Fan, Enwu Long, Lulu Cai, Qiyuan Cao, Xingwei Wu, Rongsheng Tong

**Affiliations:** ^1^ Personalized Drug Therapy Key Laboratory of Sichuan Province, School of Medicine, University of Electronic Science and Technology of China, Chengdu, China; ^2^ Department of Pharmacy, Sichuan Academy of Medical Sciences and Sichuan Provincial People’s Hospital, Chengdu, China; ^3^ West China Medical College of Sichuan University, Chengdu, China

**Keywords:** type 2 diabetes, diabetic complications, HbA1c, patient nonadherence, machine learning

## Abstract

**Purpose:** The objective of this study was to evaluate the efficacy of machine learning algorithms in predicting risks of complications and poor glycemic control in nonadherent type 2 diabetes (T2D).

**Materials and Methods:** This study was a real-world study of the complications and blood glucose prognosis of nonadherent T2D patients. Data of inpatients in Sichuan Provincial People’s Hospital from January 2010 to December 2015 were collected. The T2D patients who had neither been monitored for glycosylated hemoglobin A nor had changed their hyperglycemia treatment regimens within the last 12 months were the object of this study. Seven types of machine learning algorithms were used to develop 18 prediction models. The predictive performance was mainly assessed using the area under the curve of the testing set.

**Results:** Of 800 T2D patients, 165 (20.6%) met the inclusion criteria, of which 129 (78.2%) had poor glycemic control (defined as glycosylated hemoglobin A ≥7%). The highest area under the curves of the testing set for diabetic nephropathy, diabetic peripheral neuropathy, diabetic angiopathy, diabetic eye disease, and glycosylated hemoglobin A were 0.902 ± 0.040, 0.859 ± 0.050, 0.889 ± 0.059, 0.832 ± 0.086, and 0.825 ± 0.092, respectively.

**Conclusion:** Both univariate analysis and machine learning methods reached the same conclusion. The duration of T2D and the duration of unadjusted hypoglycemic treatment were the key risk factors of diabetic complications, and the number of hypoglycemic drugs was the key risk factor of glycemic control of nonadherent T2D. This was the first study to use machine learning algorithms to explore the potential adverse outcomes of nonadherent T2D. The performances of the final prediction models we developed were acceptable; our prediction performances outperformed most other previous studies in most evaluation measures. Those models have potential clinical applicability in improving T2D care.

## Introduction

Diabetes mellitus, characterized by persistent hyperglycemia (Li et al., 2020), is a common chronic disease. The prevalence of diabetes in China has increased rapidly from 0.67 in 1980 to 10.4% in 2013, which may be attributed to the aging of the population and changes in lifestyle (Jia et al., 2019). 10% of global health expenses is spent on diabetes (USD 760 billion) (International Diabetes Federation, 2019). Type 2 diabetes (T2D) accounts for the majority (90–95%) of individuals with diabetes mellitus (Deshpande et al., 2008; Inaishi and Saisho, 2020). Long-term hyperglycemia may lead to increased risk of diabetes-related complications including cardiovascular disease, kidney disease, retinopathy, and neuropathy (Kidanie et al., 2018). T2D and its complications harshly impact the life quality and the finances of individuals and bring about a heavy economic burden on the national health-care system (Hur et al., 2013; World Health Organization, 2016; Bui et al., 2019; Harding et al., 2019). The prevalence of these complications is generally proportional to the degree of glycemic control and the duration of diabetes (Kidanie et al., 2018). Intensive glucose control in the early stage of T2D can greatly reduce chronic complications of diabetes (Holman et al., 2008; prospective, 1995). Principles and guidelines have been used for glycemic control and preventing long-term complications for T2D (International Diabetes Federation, 2019; Jia et al., 2019; American Diabetes Association, 2020). Nevertheless, the effective treatment of T2D depends on high therapy adherence. Adherence to therapy is defined as the extent to which a person’s behavior in taking medication, monitoring of indicators, and/or following a diet corresponds with agreed recommendations from a health-care provider (García-Pérez et al., 2013). Adherence to the recommended therapy is associated with better glycemic control, fewer complications, risk reduction, and lower medical costs (Egede et al., 2012; McAdam-Marx et al., 2014; Kennedy-Martin et al., 2017; Ting et al., 2021). It is reported that nonadherence to medication among patients is common (Ting et al., 2018). Adherence to long-term therapy for chronic illnesses in developed countries averages 50%. In developing countries, the rates are even lower (World Health Organisation., 2003). A certain number of patients were found to be failing to monitor glycemia regularly nor receiving timely treatment intensification (Aujoulat et al., 2014; Reach et al., 2017; Giugliano et al., 2019; Lu et al., 2020). Early identification of potential adverse outcomes due to patient nonadherence should be an urgent priority for individualized treatment of T2D (Zarkogianni et al., 2018; Pallarés-Carratalá et al., 2019). Therefore, it was necessary to establish a prediction model that could predict the prognosis of nonadherent T2D.

“Machine learning” (ML) is also called “artificial intelligence.” The purpose of ML is to build computer systems that can adapt and learn from their experience (Kavakiotis et al., 2017). ML algorithms are commonly used to build predictive models. It can identify specific clinical variables and learn decision rules through data (Han et al., 2015; Dagliati et al., 2018; Nagaraj et al., 2019). The implementation of ML algorithms can help identify appropriate candidates for further evaluation and avoid cumbersome routine clinical steps (Handelman et al., 2018). Several studies have shown that supervised ML in medical fields can bring accurate prediction (Al'Aref et al., 2020; Meyer et al., 2018; Weiss et al., 2015; Weiss et al., 2012). However, previous studies have only applied statistics or ML models for predicting patients who may have poor adherence. Few ML models were found to predict the adverse outcomes of nonadherent T2D. In this study, we would use the local health-care systems to predict the potential adverse outcomes of nonadherent T2D.

Therefore, the objective of this work was to develop and evaluate prediction models of diabetic complications and poor glycemic control (defined as hemoglobin A1c (HbA1c) ≥7%) among nonadherent T2D patients based on ML algorithms and to identify the predictors of complications and HbA1c. Finally, it aimed to provide risk prediction tools for clinical practice.

## Materials and Methods

### Research Design and Participants

Data in this study were obtained through face-to-face investigation and the Electronic Health Medical Record System (EHRS) of Sichuan Provincial People’s Hospital. All subjects were inpatients who had been screened according to the following criteria. Patients with T2D [the World Health Organization (WHO) (1999) criteria were adopted for diagnosis of T2D] were included and would be excluded when he or she visited a medical institution within 12 months, had adjusted their treatment plan within 12 months, did not use chemicals for hypoglycemic therapy, had used traditional Chinese medicine, Chinese herbal medicine, and acupuncture to control glycemia within the last 12 months, and had liver and kidney dysfunction. The patient’s private information, such as name, home address, and contact number were hidden during the research. Informed consent forms were obtained before the investigation.

### Univariate Analysis

Univariate analysis for continuous variables was performed using t-tests, variance analysis, or the Wilcoxon signed-rank test. The categorical variables were analyzed using the chi-square test or Fisher’s exact test. *P*-values less than 0.05 (*p* < 0.05) were considered statistically significant.

### Input Variables

There were 32 input variables identified for this study, including demographic information, laboratory indicators, disease-related characteristics, medication information, and economics.

### Outcome Variables

The outcome variables were poor glycemic control and whether complications occur. In this study, HbA1c <7% was considered to be good glycemic control and ≥7% was considered to be poor glycemic control (American Diabetes Association, 2020). The complications analyzed in this study were common chronic complications of T2D.

### Variable Screening

The variables with missing values >70%, the maximum percentage of records in a single category >90%, and the maximum number of categories >95% were excluded. The minimum coefficient of variation was set to 0.1, and the minimum standard deviation was set to 0. The Pearson method was used to evaluate the correlation between input variables and outcome variables. We set the cutoff value of variable importance to 0.9 (1−*α*).

### Data Partition

The raw data were randomly split into a training set (80%) and an independent testing set (20%) by 8:2 after the variable screening. The model was built based on the training set, and the testing set was used only for the evaluation of the performance after the modeling stages. The grouping of the training set and the testing set was determined by the random seed value of the partition.

### Machine Learning Algorithms

End-to-end models were built to predict outcome variables from the input variables. The data were processed using the following ML algorithms: artificial neural network (ANN), Bayesian network (BN), chi-squared automatic interaction detector (CHAID), classification and regression tree (CRT), quick unbiased efficient statistical tree (QUEST), and discriminate (D) and ensemble (XF) models. The XF models summarized the output of the best three models (assessed by AUC) and generated their outputs based on the voting principle.

### Model Evaluation

The predictive performance of the final models was assessed by the following performance metrics: area under the receiver operating characteristic curve (AUC), negative predictive value (NPV), positive predictive value (PPV), and accuracy.

### Variable Importance

We explored the variable importance of each outcome variable derived from the best predictive model among all the tested models. Variable importance reflected the contribution of input variables to the outcome variables in specific models.

IBM SPSS Modeler 18.0 (Company Name) was used to build various models and SAS 9.21 (Company Name) was used to conduct hypothesis testing.

## Results

### Research Population

**FIGURE 1:**
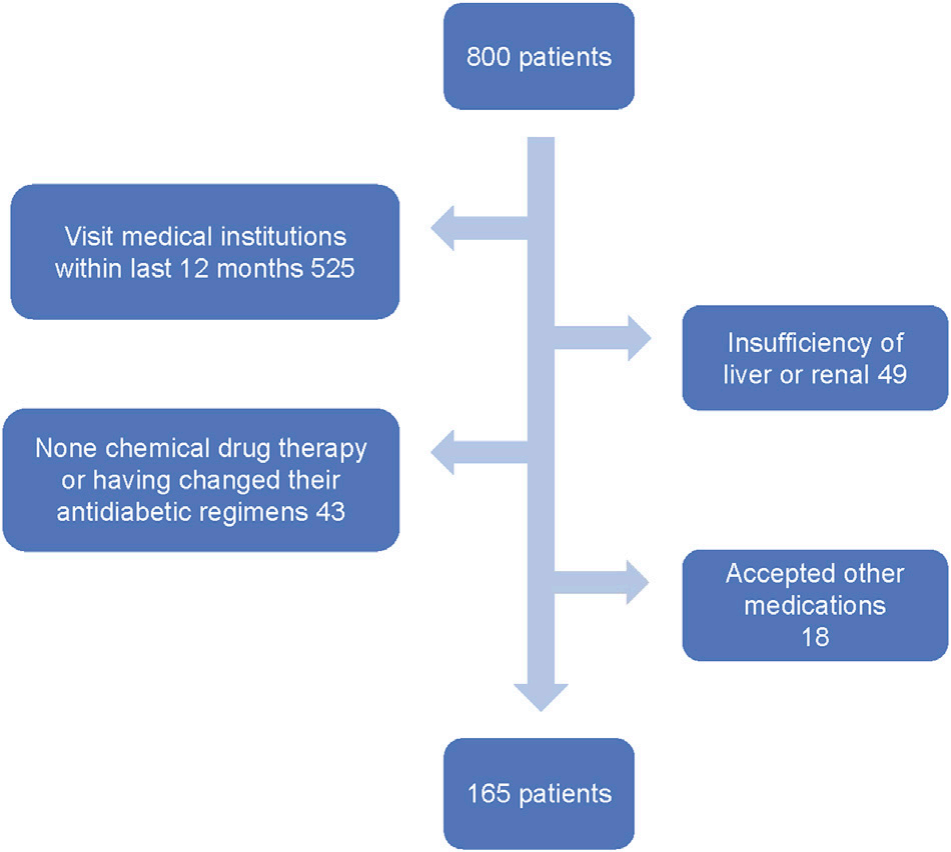
Flowchart representing the number of patients who entered the study and the detailed patient-screening process.

A total of 800 T2D patients were screened by the inclusion and exclusion criteria. 525 patients who had visited medical institutions in the past year, 49 patients who had hepatic and renal insufficiency, 43 patients who had adjusted their treatment plan and who did not use chemotherapy for hypoglycemic therapy in the last 12 months, and 18 patients who received hypoglycemic treatments other than chemical drugs in the last 12 months were excluded. The final cohort consisted of 165 patients (the screening process of patients is shown in Figure 1), including 97 male patients and 68 female patients. Seven types of complications were found in 83 cases (i.e., diabetic peripheral neuropathy (DPN), diabetic angiopathy (DA), diabetic nephropathy (DN), diabetic eye disease (DED), diabetic foot (DF), diabetic ketoacidosis (KE), and diabetic skin lesions (DD). Due to the small sample size and data imbalance, ketoacidosis (KE), diabetic skin lesions (DD), and diabetic foot (DF) were not included in this study.

### The Results of Univariate Analysis

Tables 1 and 2 list the results of univariate analysis of risk factors for complications and HbA1c in T2D patients, respectively. According to Table 1, the duration of T2D was a significant factor affecting DN (*p* < 0.0001), DPN (*p* = 0.0022), DA (*p* = 0.0015), and DED (*p* = 0.0082), and the duration of unadjusted hypoglycemic treatment was a risk factor of DN (<0.0001), DPN (<0.0001), DA (<0.0001), DED (<0.0001), and KE (<0.0284). Genetic history of diabetes was a risk factor for DPN (*p* = 0.037) and DO (*p* = 0.0189). According to Table 2, the number of hypoglycemic drugs (*p* < 0.0233) and the duration of T2D (*p* < 0.0020) were important factors affecting HbA1c. The percentage of patients with HbA1c under control declined with the prolonging of the duration of unadjusted hypoglycemic therapy.

**TABLE 1:** Univariate analysis of complications.

Characteristic		Total	DN	P	DPN	P	DA	P	DED	P	KE	P
Duration of unadjusted hypoglycemic treatment				<0.0001		<0.0001		<0.0001		<0.0001		0.0284
	1	94	8(8.51%)		12(12.77%)		6(6.38%)		2(2.13%)		1(1.06%)	
	2	37	12(32.43%)		21(56.76%)		16(43.24%)		3(8.11%)		0(0%)	
	≥3	34	19(55.88%)		32(94.12%)		20(58.82%)		10(29.41%)		3(8.82%)	
Gender				0.5902		0.5989		0.9407		0.5286		0.5110
	Male	97	21									
		39(40.21%)		25(25.77%)		10(10.31%)		3(3.09%)				
			(21.65%)									
	Female	68	17(25.00%)		25(36.76%)		17(25.00%)		5(7.35%)		1(1.47%)	
Age				0.1073		0.2169		0.3698		0.4278		0.0664
	≤ 45	12	2(16.67%)		5(41.67%)		5(41.67%)		1(8.33%)		1(8.33%)	
	45–55 (included)	36	6(16.67%)		12(33.33%)		9(25.00%)		3(8.33%)		2(1.20%)	
	55–65 (included)	34	6(17.65%)		11(32.35%)		10(29.41%)		2(5.88%)		0(0%)	
	65–75 (included)	38	11(28.95%)		14(36.84%)		6(15.79%)		3(7.89%)		1(2.63%)	
	> 75	45	13(28.89%)		22(48.89%)		12(26.67%)		6(13.33%)		0(0%)	
Duration of T2D				<0.0001		0.0022		0.0015		0.0082		0.7701
	1–2 (included) years	22	0(0%)		4(18.18%)		2(9.09%)		0(0%)		1(4.55%)	
	2–5 (included) years	22	3(13.64%)		5(22.73%)		2(9.09%)		1(4.55%)		0(0%)	
	5–10 (included) years	71	13(18.31%)		28(39.44%)		19(26.76%)		5(7.04%)		2(2.82%)	
	> 10 years	50	22(44.00%)		27(54.00%)		19(38.00%)		9(18.00%)		1(2.00%)	
Hereditary history				0.5824		0.037		0.1291		0.0189		0.714
	No	113	26(23.01%)		39(34.51%)		26(23.01%)		7(6.19%)		2(1.77%)	
	Yes	37	10(27.03%)		20(54.05%)		13(35.14%)		7(18.92%)		1(2.70%)	

Data are number (%).

The duration of T2D and the duration of unadjusted hypoglycemic treatment were significant risk factors affecting DN, DPN, DA, and DED.

The genetic history of diabetes was a risk factor for DPN and DO.

DN, diabetic nephropathy; DPN, diabetic peripheral neuropathy; DA, diabetic angiopathy; DED, diabetic eye disease; KE, ketoacidosis.

**TABLE 2:** Univariate analysis of HbA1c.

Characteristic		Number of patients	Number of patients with HbA1c <7%	Number of patients with HbA1c ≥7%	*p*-value
Duration of unadjusted hypoglycemic treatment					0.6462
	1 (included)–2 years	84	21(25.00%)	63	
	2 (included)–3 years	35	10(28.57%)	25	
	≥3 years	32	4(12.50%)	28	
Gender					0.3701
	Male	88	17(19.32%)	71	
	Female	63	18(28.57%)	45	
Age					0.2084
	≤45	11	3(27.27%)	8	
	45–55 (included)	30	8(26.67%)	22	
	55–65 (included)	31	4(12.90%)	27	
	65–75 (included)	37	11(29.73%)	26	
	>75	42	9(21.43%)	33	
Duration of T2D					0.0020
	1–2 (included) years	21	9(42.86%)	12	
	2–5 (included) years	18	7(38.89%)	11	
	5–10 (included) years	63	11(17.46%)	52	
	> 10 years	49	8(16.33%)	41	
Hereditary history					0.0753
	No	101	30(29.70%)	71	
	Yes	37	5(13.51%)	32	
Number of hypoglycemic drugs					0.0233
	One	55	21(38.18%)	34	
	Two	63	9(14.29%)	54	
	Three	27	5(18.52%)	22	
	Four	5	0(0%)	5	
	Five	1	0(0%)	1	

The number of hypoglycemic drugs and the duration of T2D were risk factors of HbA1c.

HbA1c, hemoglobin A1c.

### The Results of Variable Screening

Among the total of 32 input variables, 18 were excluded due to the low correlation with the characteristics of the outcome variable, and five were excluded due to data imbalance. There were nine input variables and five outcome variables that were retained for the development of the final models. The input variables were age, duration of diabetes (≥1 year), duration of unadjusted hypoglycemic treatment (≥1 year), number of insulin species, total cost (total expenditure during hospitalization) of hypoglycemic drugs, and number of hypoglycemic drugs (which were computed as continuous variables) and gender, genetic history of diabetes, and dyslipidemia (which were computed as categorical variables). The outcome variables included the onset of DPN, DA, DED, and DN and the control status of glycemia.

### The Results of Model Prediction

**FIGURE 2:**
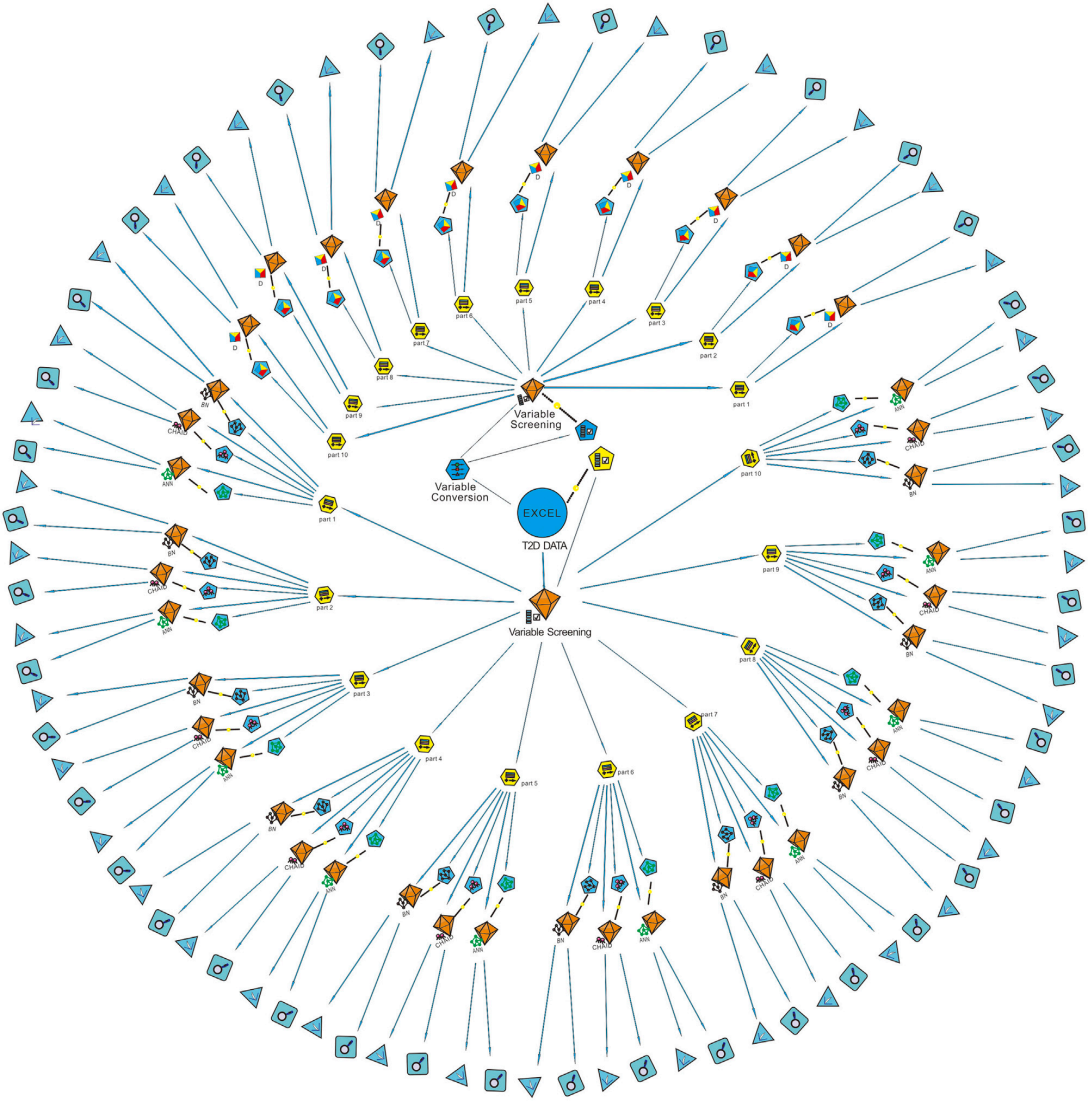
Modeling steps of diabetic eye disease (DED). The “variable screening” node was used for data preprocessing after the “T2D data” were imported. Since the D model can only identify continuous variables, the “variable conversion” node was used to convert categorical variables into continuous variables. The “partition” node was used to divide the dataset into a training set and a testing set randomly by 8:2. Ten partitions were generated for each dataset by modifying the random seed value. Machine learning algorithms of BN, CHAID, ANN, and D were used for modeling after partition. Finally, the ROC curve and confusion matrix of each model was output through the two nodes at the end of the data stream. AUC obtained from the confusion matrix of the testing set was used for model verification. T2D, type 2 diabetes; Part, partition; D, discriminate; BN, Bayesian network; ANN, artificial neural network; CHAID, chi-squared automatic interaction detector.

**FIGURE 3:**
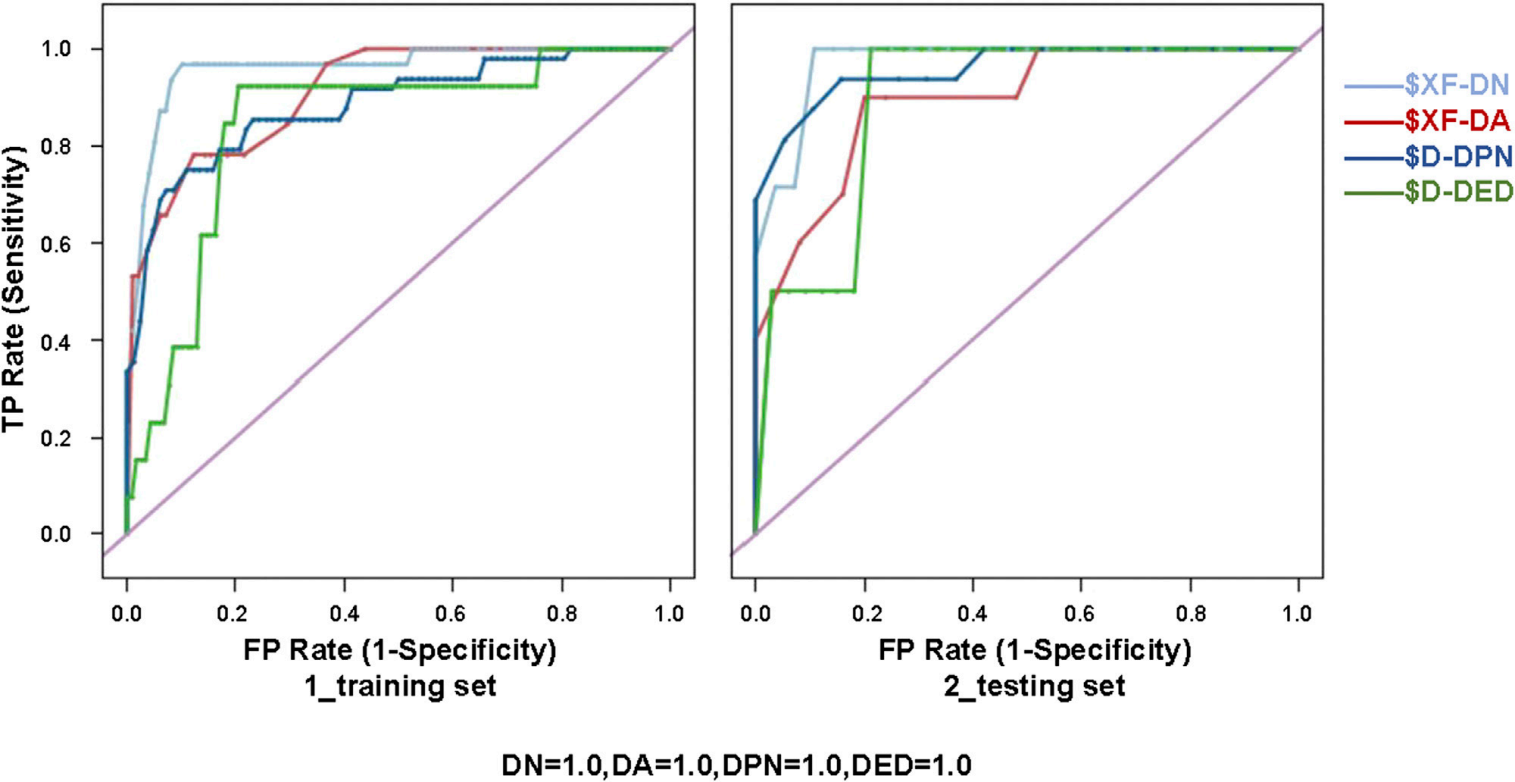
ROC curve for the model with the highest AUC of each diabetic complication (i.e., XF of DN, XF of DA, D of DPN, and D of DED) for the training (80%) and testing (20%) sets. XF, ensemble model; D, discriminate; DN, diabetic nephropathy; DA, diabetic angiopathy; DPN, diabetic peripheral neuropathy; DED, diabetic eye disease; ROC, receiver operating characteristic.

Sixteen best-performing algorithms with the highest AUCs were selected for modeling of four complications, and two best-performing algorithms were selected for HbA1c. Ten independent replicate results were generated for each model by changing the data split of a dataset. This was achieved by modifying random seeds of the “partition” node. A total of 180 models were obtained. The modeling steps of DED are shown in Figure 2, and the ROC curve for the model with the highest AUC of each complication is shown in Figure 3.

The PPV, NPV, accuracy, and AUC for different ML algorithms by the testing set are shown in Table 3. Among the 18 evaluated models, most models performed well. XF performed best among all the predictive models of DN and DA, with AUCs of 0.902 ± 0.040 and 0.889 ± 0.059. D performed best among all the models of DPN and DED, with AUCs of 0.859 ± 0.050 and 0.832 ± 0.086. The best model for HbA1c was considered to be BN, with the highest AUC of 0.825 ± 0.092.

**TABLE 3:** Predictive performance of different models using the testing (i.e., 20%) set.

Outcome variables	Model types	AUC	Accuracy	PPV	NPV
DN	XF	0.902 ± 0.040	0.862 ± 0.054	0.699 ± 0.149	0.917 ± 0.050
	CHAID	0.699 ± 0.074	0.699 ± 0.067	0.331 ± 0.158	0.780 ± 0.068
	BN	0.744 ± 0.078	0.916 ± 0.057	0.930 ± 0.091	0.914 ± 0.066
	D	0.823 ± 0.055	0.720 ± 0.063	0.459 ± 0.132	0.895 ± 0.064
DPN	XF	0.847 ± 0.081	0.783 ± 0.080	0.642 ± 0.123	0.882 ± 0.073
	CHAID	0.787 ± 0.081	0.757 ± 0.054	0.680 ± 0.143	0.807 ± 0.070
	QUEST	0.720 ± 0.060	0.766 ± 0.056	0.716 ± 0.186	0.805 ± 0.057
	D	0.859 ± 0.050	0.843 ± 0.038	0.775 ± 0.092	0.885 ± 0.055
DA	XF	0.889 ± 0.059	0.851 ± 0.051	0.684 ± 0.129	0.899 ± 0.045
	CHAID	0.764 ± 0.087	0.769 ± 0.049	0.481 ± 0.229	0.842 ± 0.066
	CRT	0.797 ± 0.068	0.802 ± 0.058	0.671 ± 0.207	0.836 ± 0.064
	D	0.825 ± 0.070	0.808 ± 0.065	0.568 ± 0.150	0.907 ± 0.056
DED	ANN	0.725 ± 0.142	0.812 ± 0.091	0.083 ± 0.180	0.864 ± 0.080
	CHAID	0.818 ± 0.161	0.875 ± 0.053	0.523 ± 0.346	0.916 ± 0.050
	BN	0.749 ± 0.179	0.978 ± 0.031	0.867 ± 0.322	0.984 ± 0.028
	D	0.832 ± 0.086	0.799 ± 0.055	0.328 ± 0.156	0.989 ± 0.025
HbA1c	ANN	0.604 ± 0.103	0.760 ± 0.094	0.375 ± 0.460	0.825 ± 0.089
	BN	0.825 ± 0.092	0.728 ± 0.083	0.417 ± 0.180	0.840 ± 0.120

Data are mean ± SD.

XF, ensemble model; ANN, artificial neural network; CRT, classification and regression tree; QUEST, quick unbiased efficient statistical tree; D, discriminate; BN, Bayesian network; DN, diabetic nephropathy; DPN, diabetic peripheral neuropathy; DA, diabetic angiopathy; DED, diabetic eye disease; HbA1c, glycosylated hemoglobin A; AUC, area under the receiver operating characteristic curve; PPV, positive predictive value; NPV, negative predictive value.

### Variable Importance

**FIGURE 4:**
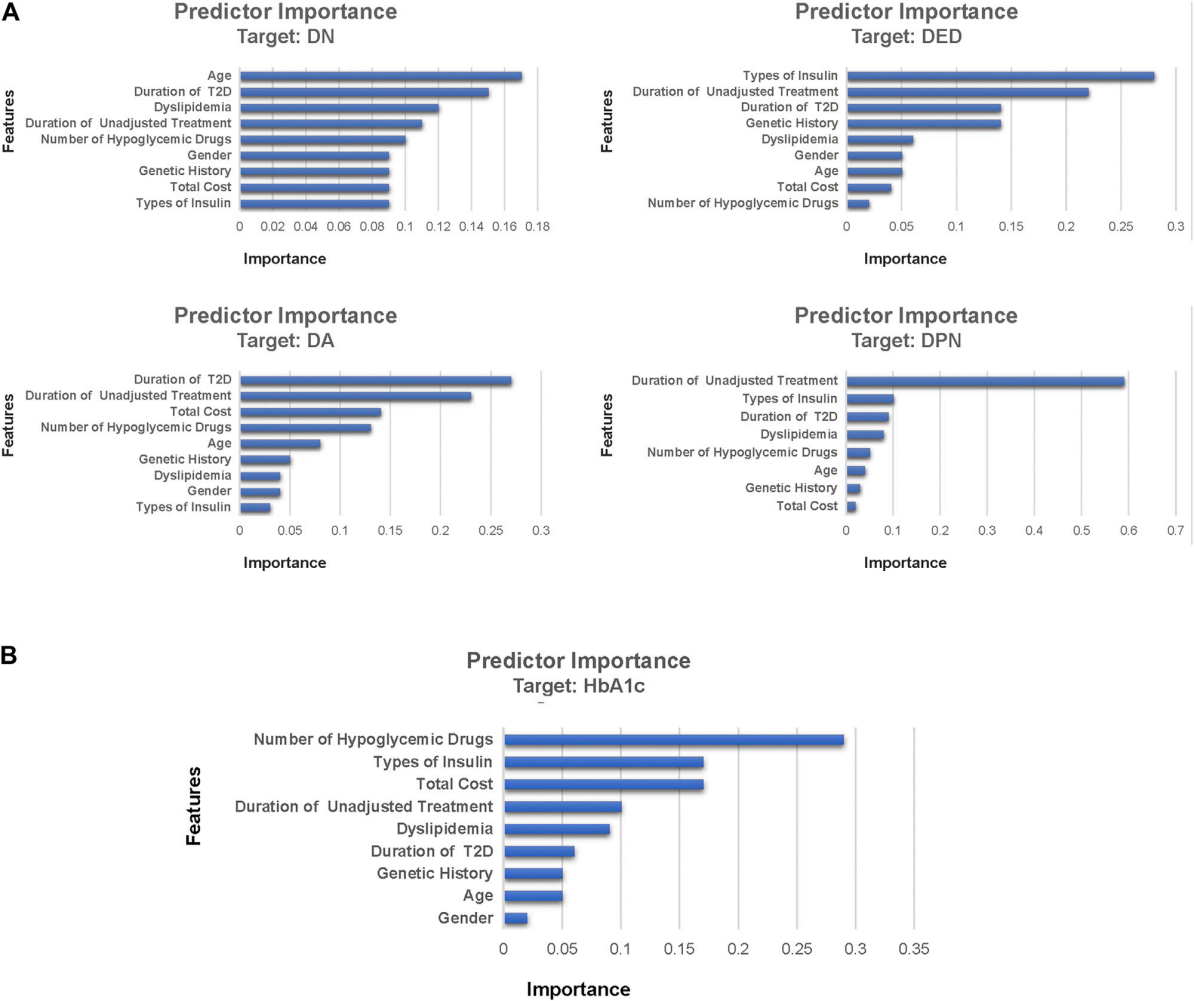
Feature importance of DN, DA, DED, DPN, and HbA1c derived from machine learning algorithms. Part **(A)** was the feature importance of diabetic complications and part (**B**) was the feature importance of HbA1c. Feature importance describes the relative importance of input variables for a single outcome variable in the supervised models. DN, diabetic nephropathy; DPN, diabetic peripheral neuropathy; DA, diabetic angiopathy; DED, diabetic eye disease; HbA1c, glycosylated hemoglobin A.

Figure 4 shows the variable importance of DN, DA, DED, DPN, and HbA1c derived from the best-performing ML algorithms. It also shows the relative importance of the variables with the top three most important variables of complications being the duration of T2D, the duration of unadjusted hypoglycemic treatment, and types of insulin. The top three most important variables of HbA1c were the number of hypoglycemic drugs, types of insulin, and total cost. The most important variables of DN, DA, DED, and DPN were age, duration of T2D, types of insulin, and duration of unadjusted hypoglycemic treatment, respectively. A novel predictive variable, the duration of unadjusted hypoglycemic treatment (during this time, the patient’s hypoglycemic treatment regimen remains unchanged, and relevant follow-up monitoring has not been performed), of T2D was identified from this study. We can predict the probability of complications in T2D patients through the duration of the hypoglycemic regimen.

## Discussion

### Results and Discussion

This study employed ML algorithms to screen for cases likely to have diabetic complications and poor glycemic control among nonadherent T2D patients and provided potential risk prediction tools for both outcomes. Eighteen models were evaluated, and the risk factors for complications and poor glycemic control were identified, with the most important risk factors being the duration of T2D, the duration of unadjusted hypoglycemic treatment, types of insulin, number of hypoglycemic drugs, and total cost of hypoglycemic therapy. The prediction models we established in this study obtained acceptable performances. According to previous reports, under-monitoring and delay of treatment are major challenges to diabetes management (Ross et al., 2011; Khunti et al., 2013; Khunti et al., 2016). The findings of this study are important because early screening may strengthen glycemic control and reduce the risk of diabetic complications through timely monitoring of glycemia and treatment intensification (Holman et al., 2008; Colagiuri and Davies, 2009; Griffin et al., 2011).

ML algorithms have been widely used in medical fields recently (Cichosz et al., 2015; Kavakiotis et al., 2017; Contreras and Vehi, 2018; Lan et al., 2019). It is the key technology of big data analysis, which provides new ways for clinicians to solve medical problems (Hui et al., 2016). Recent advances in ML algorithms have improved the accuracy of diagnosis and prediction in outcomes, in some cases even surpassing the performance of clinicians (Beam and Kohane, 2018). ML-based prediction models for classification or prediction of future health states are being developed (Emanuel and Wachter, 2019).

A large number of studies have reported on the prediction model of diabetic complications and glycemia. In the study of Hsin-Yi Tsao et al., data mining techniques were used to create prediction models of diabetic retinopathy, with results that indicated that insulin therapy and duration of diabetes are the most important risk factors of diabetic retinopathy, which was consistent with this study (Tsao et al., 2018). Compared with previous research, our study also found a new risk factor, the duration of unadjusted hypoglycemic treatment, for DED. Konstantia Zarkogianni et al. developed a risk prediction model for T2D cardiovascular complication (Zarkogianni et al., 2018). As with most predictive models, the prediction results are difficult to interpret. In our study, the prediction results were interpretable due to the use of decision trees. Dennis H Murphree et al. built several ML models to predict good HbA1c control (<7.0%) among T2D patients, which showed the potential for applying ML to solve problems in medical fields (Tsao et al., 2018). Consistent with prior research studies (Murphree et al., 2018; Tsao et al., 2018; Zarkogianni et al., 2018; Aminian et al., 2020), the findings of this study showed high AUCs.

Previous studies have explored the characteristics of patients with medication nonadherence from different perspectives. A systematic review analyzed the relationship between medication nonadherence and the health outcomes in the elderly (Walsh et al., 2019) and showed that medication nonadherence may be significantly associated with all-cause hospitalization and mortality in old people (Walsh et al., 2019). Instead of the senior group, the subjects of our study were T2D patients, and the prognosis of T2D we predicted was HbA1c and diabetic complications. In a cross-sectional study, the author explored the main predictors of poor adherence among T2D patients (Demoz et al., 2020). Another study, a previous article by Dr. Wu, assessed multiple ML algorithms and predicted the medication nonadherence risks of patients with T2D (Wu et al., 2020). The two articles above were studies on the influencing factors of medication nonadherence, and the predicted outcome is the compliance of patients. Both articles are quite different from our work. In our study, statistical and ML methods were used to predict risk factors of HbA1c and potential complications of nonadherent T2D. The research method and outcomes were quite different. New research ideas were provided for the influencing factors and prediction models of T2D progression.

### Study Limitations and Strengths

This study had some strengths. Instead of a generic cohort, a highly specific one, nonadherent T2D patients, was used. This was the first study to use ML models to explore the health outcomes of nonadherent T2D. Besides, the internal validation of these models was conducted using the following method. The raw data were randomly grouped ten times by modifying the seed value of the “partition.” In this way, independently repeated experiments were conducted, and the bias that may occur when datasets are randomly grouped was avoided. This method is also better than bootstrapping (Milea et al., 2020), which may increase the weight of some data. Moreover, the dataset we used for prediction contained clinical information that has not been studied before, which is the duration of unadjusted hypoglycemic treatment.

However, there were some notable limitations to this study. This was a single-center, small-sample study, and the performance of the final models was not compared with that of the established clinical reference tools, which limits the reliability of the verification results. Nevertheless, the influencing factors were analyzed through conventional statistical calculations, and the results of the univariate analysis were consistent with the prediction models. In the future, a large-scale, forward-looking, and multicenter study is needed for further external validation.

## Conclusion

Among the nonadherent T2D patients, duration of T2D and duration of unadjusted hypoglycemic treatment were the key risk factors of diabetic complications. The number of hypoglycemic drugs was the key risk factor of glycemic control. The enhancement of medication compliance in patients with T2D and the strengthening of blood glucose monitoring and control are beneficial to delaying the occurrence and development of T2D complications and provide evidence support for the individualized management of T2D. In this study, after the validation and screening of prediction models, the final models derived in this study may be clinically useful for patients with T2D and health-care professionals, including general practitioners and endocrinologists. The findings of this study may provide evidence of the potential adverse outcomes based on the current health situation, help to improve the treatment adherence of T2D patients, and reduce the burden of individuals and national health-care systems.

## Data Availability

The raw data supporting the conclusions of this article will be made available by the authors, without undue reservation.

## References

[B3] Al'Aref S. J. Maliakal G. Singh G. van Rosendael A. R. Ma X. Xu Z. (2020). Machine Learning of Clinical Variables and Coronary Artery Calcium Scoring for the Prediction of Obstructive Coronary Artery Disease on Coronary Computed Tomography Angiography: Analysis from the CONFIRM Registry. Eur. Heart J. 41, 359–367. 10.1093/eurheartj/ehz565 31513271 PMC7849944

[B2] American Diabetes Association (2020). 6. Glycemic Targets: Standards of Medical Care in Diabetes-2020. Diabetes Care 43, S66–S76. 10.2337/dc20-S006 31862749

[B4] Aminian A. Zajichek A. Arterburn D. E. Wolski K. E. Brethauer S. A. Schauer P. R. (2020). Predicting 10-Year Risk of End-Organ Complications of Type 2 Diabetes with and without Metabolic Surgery: A Machine Learning Approach. Dia Care 43, 852–859. 10.2337/dc19-2057 PMC7646205 32029638

[B5] Aujoulat I. Jacquemin P. Darras E. Rietzschel E. Wens J. Hermans M. (2014). Factors Associated with Clinical Inertia: an Integrative Review. Amep 5, 141–147. 10.2147/AMEP.S59022 PMC4028485 24868181

[B6] Beam A. L. Kohane I. S. (2018). Big Data and Machine Learning in Health Care. JAMA 319, 1317–1318. 10.1001/jama.2017.18391 29532063

[B7] Bui H. D. T. Jing X. Lu R. Chen J. Ngo V. Cui Z. (2019). Prevalence of and Factors Related to Microvascular Complications in Patients with Type 2 Diabetes Mellitus in Tianjin, China: a Cross-Sectional Study. Ann. Transl. Med. 7, 325. 10.21037/atm.2019.06.08 31475195 PMC6694230

[B8] Cichosz S. L. Johansen M. D. Hejlesen O. (2015). Toward Big Data Analytics. J. Diabetes Sci. Technol. 10, 27–34. 10.1177/1932296815611680 26468133 PMC4738225

[B9] Colagiuri S. Davies D. (2009). The Value of Early Detection of Type 2 Diabetes. Curr. Opin. Endocrinol. Diabetes Obes. 16, 95–99. 10.1097/MED.0b013e328329302f 19276801

[B10] Contreras I. Vehi J. (2018). Artificial Intelligence for Diabetes Management and Decision Support: Literature Review. J. Med. Internet Res. 20, e10775. 10.2196/10775 29848472 PMC6000484

[B11] Dagliati A. Marini S. Sacchi L. Cogni G. Teliti M. Tibollo V. (2018). Machine Learning Methods to Predict Diabetes Complications. J. Diabetes Sci. Technol. 12, 295–302. 10.1177/1932296817706375 28494618 PMC5851210

[B12] Demoz G. T. Wahdey S. Bahrey D. Kahsay H. Woldu G. Niriayo Y. L. Predictors of Poor Adherence to Antidiabetic Therapy in Patients with Type 2 Diabetes: a Cross-Sectional Study Insight from Ethiopia. Diabetol. Metab. Syndr. 2020;12:62. 10.1186/s13098-020-00567-7 32695232 PMC7364580

[B13] Deshpande A. D. Harris-Hayes M. Schootman M. (2008). Epidemiology of Diabetes and Diabetes-Related Complications. Phys. Ther. 88 (11), 1254–1264. 10.2522/ptj.20080020 18801858 PMC3870323

[B14] Egede L. E. Gebregziabher M. Dismuke C. E. Lynch C. P. Axon R. N. Zhao Y. (2012). Medication Nonadherence in Diabetes: Longitudinal Effects on Costs and Potential Cost Savings from Improvement. Diabetes Care 35, 2533–2539. 10.2337/dc12-0572 22912429 PMC3507586

[B15] Emanuel E. J. Wachter R. M. (2019). Artificial Intelligence in Health Care. JAMA 321, 2281–2282. 10.1001/jama.2019.4914 31107500

[B16] García-Pérez L.-E. Álvarez M. Dilla T. Gil-Guillén V. Orozco-Beltrán D. (2013). Adherence to Therapies in Patients with Type 2 Diabetes. Diabetes Ther. 4(2):175–194. 10.1007/s13300-013-0034-y 23990497 PMC3889324

[B17] Giugliano D. Maiorino M. I. Bellastella G. Esposito K. (2019). Clinical Inertia, Reverse Clinical Inertia, and Medication Non-adherence in Type 2 Diabetes. J. Endocrinol. Invest. 42, 495–503. 10.1007/s40618-018-0951-8 30291589

[B18] Griffin S. J. Borch-Johnsen K. Davies M. J. Khunti K. Rutten G. E. Sandbæk A. (2011). Effect of Early Intensive Multifactorial Therapy on 5-year Cardiovascular Outcomes in Individuals with Type 2 Diabetes Detected by Screening (ADDITION-Europe): a Cluster-Randomised Trial. The Lancet 378, 156–167. 10.1016/S0140-6736(11)60698-3 PMC3136726 21705063

[B19] Han D. Wang S. Jiang C. Jiang X. Kim H.-E. Sun J. (2015). Trends in Biomedical Informatics: Automated Topic Analysis of JAMIA Articles. J. Am. Med. Inform. Assoc. 22, 1153–1163. 10.1093/jamia/ocv157 26555018 PMC5009912

[B20] Handelman G. S. Kok H. K. Chandra R. V. Razavi A. H. Lee M. J. Asadi H. (2018). eDoctor: Machine Learning and the Future of Medicine. J. Intern. Med. 284, 603–619. 10.1111/joim.12822 30102808

[B21] Harding J. L. Pavkov M. E. Magliano D. J. Shaw J. E. Gregg E. W. (2019). Global Trends in Diabetes Complications: a Review of Current Evidence. Diabetologia 62 (1), 3–16. 10.1007/s00125-018-4711-2 30171279

[B22] Holman R. R. Paul S. K. Bethel M. A. Matthews D. R. Neil H. A. W. (2008). 10-year Follow-Up of Intensive Glucose Control in Type 2 Diabetes. N. Engl. J. Med. 359 (15), 1577–1589. 10.1056/NEJMoa0806470 18784090

[B24] Hui H. Zheng P. Zhang Y. (2016). Medical Big Data Research Facing on Opportunities and Developing Trends. Chin. Health Qual. Manage. 23, 91–93. 10.23883/ijrter.conf.20171201.060.brkrh

[B25] Hur J. Sullivan K. A. Callaghan B. C. Pop-Busui R. Feldman E. L. (2013). Identification of Factors Associated with Sural Nerve Regeneration and Degeneration in Diabetic Neuropathy. Diabetes Care 36, 4043–4049. 10.2337/dc12-2530 24101696 PMC3836098

[B27] Inaishi J. Saisho Y. (2020). Beta-Cell Mass in Obesity and Type 2 Diabetes, and its Relation to Pancreas Fat: A Mini-Review. Nutrients 12 (12), 3846. 10.3390/nu12123846 PMC7766247 33339276

[B26] International Diabetes Federation (2019). IDF Diabetes Atlas. 9. Brussels. International Diabetes Federation. Available at: https://www.diabetesatlas.org/en/# ( (Accessed February 5, 2021).

[B28] Jia W. Weng J. Zhu D. Ji L. Lu J. Zhou Z. (2019). Standards of Medical Care for Type 2 Diabetes in China 2019. Diabetes Metab. Res. Rev. 35, e3158. 10.1002/dmrr.3158 30908791

[B29] Kavakiotis I. Tsave O. Salifoglou A. Maglaveras N. Vlahavas I. Chouvarda I. (2017). Machine Learning and Data Mining Methods in Diabetes Research. Comput. Struct. Biotechnol. J. 15 (C), 104–116. 10.1016/j.csbj.2016.12.005 28138367 PMC5257026

[B31] Kennedy-Martin T. Boye K. Peng X. (2017). Cost of Medication Adherence and Persistence in Type 2 Diabetes Mellitus: a Literature Review. Ppa 11, 1103–1117. 10.2147/PPA.S136639 28721024 PMC5501621

[B33] Khunti K. Nikolajsen A. Thorsted B. L. Andersen M. Davies M. J. Paul S. K. (2016). Clinical Inertia with Regard to Intensifying Therapy in People with Type 2 Diabetes Treated with Basal Insulin. Diabetes Obes. Metab. 18, 401–409. 10.1111/dom.12626 26743666 PMC5067688

[B32] Khunti K. Wolden M. L. Thorsted B. L. Andersen M. Davies M. J. (2013). Clinical Inertia in People with Type 2 Diabetes: a Retrospective Cohort Study of More Than 80,000 People. Diabetes Care 36, 3411–3417. 10.2337/dc13-0331 23877982 PMC3816889

[B34] Kidanie B. B. Alem G. Zeleke H. Gedfew M. Edemealem A. Andualem A. (2018). Determinants of Diabetic Complication Among Adult Diabetic Patients in Debre Markos Referral Hospital, Northwest Ethiopia, 2018: Unmatched Case Control StudyUnmatched Case Control Study. Dmso 13, 237–245. 10.2147/DMSO.S237250 PMC7007775 32099430

[B35] Lan X. Wei R. Cai H. Guo Y. Hou M. Xing L. (2019). Application of Machine Learning Algorithms in the Medical Field. Med. Health equipment 40, 101–105.

[B36] Li W. Lin L. Yan D. Jin Y. Xu Y. Li Y. (2020). Application of a Pseudotargeted MS Method for the Quantification of Glycated Hemoglobin for the Improved Diagnosis of Diabetes Mellitus. Anal. Chem. 92, 3237–3245. 10.1021/acs.analchem.9b05046 31961136

[B37] Lu J. Ma X. Shen Y. Wu Q. Wang R. Zhang L. (2020). Time in Range Is Associated with Carotid Intima-Media Thickness in Type 2 Diabetes. Diabetes Tech. Ther. 22, 72–78. 10.1089/dia.2019.0251 31524497

[B38] McAdam-Marx C. Bellows B. K. Unni S. Wygant G. Mukherjee J. Ye X. (2014). Impact of Adherence and Weight Loss on Glycemic Control in Patients with Type 2 Diabetes: Cohort Analyses of Integrated Medical Record, Pharmacy Claims, and Patient-Reported Data. Jmcp 20, 691–700. 10.18553/jmcp.2014.20.7.691 24967522 PMC10437951

[B39] Meyer A. Zverinski D. Pfahringer B. Kempfert J. Kuehne T. Sündermann S. H. (2018). Machine Learning for Real-Time Prediction of Complications in Critical Care: a Retrospective Study. Lancet Respir. Med. 6, 905–914. 10.1016/S2213-2600(18)30300-X 30274956

[B40] Milea D. Najjar R. P. Jiang Z. Ting D. Vasseneix C. Xu X. (2020). Artificial Intelligence to Detect Papilledema from Ocular Fundus Photographs. N. Engl. J. Med. 382, 1687–1695. 10.1056/nejmoa1917130 32286748

[B41] Murphree D. H. Arabmakki E. Ngufor C. Storlie C. B. McCoy R. G. (2018). Stacked Classifiers for Individualized Prediction of Glycemic Control Following Initiation of Metformin Therapy in Type 2 Diabetes. Comput. Biol. Med. 103, 109–115. 10.1016/j.compbiomed.2018.10.017 30347342 PMC6279555

[B42] Nagaraj S. B. Sidorenkov G. Boven J. F. M. Denig P. (2019). Predicting Short‐ and Long‐term Glycated Haemoglobin Response after Insulin Initiation in Patients with Type 2 Diabetes Mellitus Using Machine‐learning Algorithms. Diabetes Obes. Metab. 21, 2704–2711. 10.1111/dom.13860 31453664 PMC6899933

[B43] Pallarés-Carratalá V. Bonig-Trigueros I. Palazón-Bru A. Esteban-Giner M. J. Gil-Guillén V. F. Giner-Galvañ V. (2019). Clinical Inertia in Hypertension: a New Holistic and Practical Concept within the Cardiovascular Continuum and Clinical Care Process. Blood Press. 28, 217–228. 10.1080/08037051.2019.1608134 31023106

[B44] prospective U. K. (1995). U.K. Prospective Diabetes Study 16. Overview of 6 years' Therapy of Type II Diabetes: a Progressive Disease. U.K. Prospective Diabetes Study Group. Diabetes 44 (11), 1249–1258. 7589820

[B45] Reach G. Pechtner V. Gentilella R. Corcos A. Ceriello A. (2017). Clinical Inertia and its Impact on Treatment Intensification in People with Type 2 Diabetes Mellitus. Diabetes Metab. 43, 501–511. 10.1016/j.diabet.2017.06.003 28754263

[B46] Ross S. A. Tildesley H. D. Ashkenas J. (2011). Barriers to Effective Insulin Treatment: the Persistence of Poor Glycemic Control in Type 2 Diabetes. Curr. Med. Res. Opin. 27, 13–20. 10.1185/03007995.2011.621416 21942467

[B47] Ting C. Y. Ahmad Zaidi Adruce S. Hassali M. A. Ting H. Lim C. J. Ting R. S.-K. (2018). Effectiveness and Sustainability of a Structured Group-Based Educational Program (MEDIHEALTH) in Improving Medication Adherence Among Malay Patients with Underlying Type 2 Diabetes Mellitus in Sarawak State of Malaysia: Study Protocol of a Randomized Controlled Trial. Trials 19 (1), 310. 10.1186/s13063-018-2649-9 29871651 PMC5989376

[B48] Ting C. Y. Ahmad Zaidi Adruce S. Lim C. J. Abd Jabar A. H. A. Ting R. S.-K. Ting H. (2021). Effectiveness of a Pharmacist-Led Structured Group-Based Intervention in Improving Medication Adherence and Glycaemic Control Among Type 2 Diabetes Mellitus Patients: A Randomized Controlled Trial. Res. Soc. Administrative Pharm. 17, 344–355. 10.1016/j.sapharm.2020.03.026 32327398

[B49] Tsao H.-Y. Chan P.-Y. Su E. C.-Y. (2018). Predicting Diabetic Retinopathy and Identifying Interpretable Biomedical Features Using Machine Learning Algorithms. BMC Bioinformatics 19, 283. 10.1186/s12859-018-2277-0 30367589 PMC6101083

[B50] Walsh C. A. Cahir C. Tecklenborg S. Byrne C. Culbertson M. A. Bennett K. E. (2019). The Association between Medication Non‐adherence and Adverse Health Outcomes in Ageing Populations: A Systematic Review and Meta‐analysis. Br. J. Clin. Pharmacol. 85 (11), 2464–2478. 10.1111/bcp.14075 31486099 PMC6848955

[B52] Weiss J. Kuusisto F. Boyd K. Liu J. Page D. (2015). Machine Learning for Treatment Assignment: Improving Individualized Risk Attribution. AMIA Annu. Symp. Proc. 2015, 1306–1315. 26958271 PMC4765638

[B51] Weiss J. C. Natarajan S. Peissig P. L. Mccarty C. A. Page D. (2012). Machine Learning for Personalized Medicine: Predicting Primary Myocardial Infarction from Electronic Health Records. AIMag 33, 33–45. 10.1609/aimag.v33i4.2438 PMC4211289 25360347

[B1] World Health Organisation. Adherence to Long Term Therapies; Evidence for Action. 2003 Available at: http://www.who.int/chp/knowledge/publications/adherencefullreport.pdf?ua=1 (Accessed 6 Nov 2015).

[B53] World Health Organization (2016). Global Report on Diabetes: World Health Organization. Available at: http://apps.who.int/iris/bitstream/10665/204871/1/ 9789241565257_eng.pdf (Accessed March 18, 2017)

[B54] Wu X.-W. Yang H.-B. Yuan R. Long E.-W. Tong R.-S. (2020). Predictive Models of Medication Non-adherence Risks of Patients with T2D Based on Multiple Machine Learning Algorithms. BMJ Open Diab Res. Care 8 (1), e001055. 10.1136/bmjdrc-2019-001055 PMC7064141 32156739

[B55] Zarkogianni K. Athanasiou M. Thanopoulou A. C. Nikita K. S. (2018). Comparison of Machine Learning Approaches toward Assessing the Risk of Developing Cardiovascular Disease as a Long-Term Diabetes Complication. IEEE J. Biomed. Health Inform. 22, 1637–1647. 10.1109/JBHI.2017.2765639 29990007

